# Data archiving and availability in an era of open science

**DOI:** 10.1107/S2052252516020340

**Published:** 2017-01-01

**Authors:** Edward N. Baker

**Affiliations:** a School of Biological Sciences, University of Auckland, School of Biological Sciences, Private Bag 92-019, Auckland, New Zealand

**Keywords:** raw diffraction data, data archiving, open science

## Abstract

The importance of preserving and making available the original experimental data underlying biological structural models is discussed, both for crystallography, where the raw data images pose particular challenges, and for other structure determination techniques.

The current moves to increasing openness in science have both philosophical and scientific rationales, and carry great potential benefits for science. The belief that the results of publicly funded research should be freely available to all is only part of this. Science itself is a form of international cultural heritage, and can best develop if the ideas it brings are spread as widely as possible. Open-access publishing, as exemplified by this journal, is one means by which this can be done.

Of equal importance, however, is the need to preserve the underlying experimental data, preferably in a manner that makes them available to others. This enables results to be validated, re-evaluated or extended, increasing the value of the original work and opening possibilities for new directions. Crystallography has an inbuilt advantage here in that it is data-rich and the data are readily stored in electronic form. (This does not apply to the original biological or chemical samples, unfortunately, or to crystals, but that is another story). We have also been extraordinarily fortunate, since the earliest days of structural biology, in having scientists within our discipline with the vision to see the importance of archiving the structural and diffraction data, to preserve them, organize and annotate them and make them freely available (Berman *et al.*, 2016 ▸). The Protein Data Bank (PDB) and its successor, the worldwide PDB (wwPDB), which is curated by its United States, European and Japanese partners (Berman *et al.*, 2003 ▸), is a wonderful resource today, well managed and forward looking.

Today, more than 120 000 macromolecular structures determined by crystallography are archived in the wwPDB and are joined by some 11 000 determined by NMR and 1100 by cryo-EM. The latter bring different kinds of data to be archived, and require different forms of validation, which are currently being worked through by expert taskforces for implementation within the wwPDB. Structures determined by cryo-EM, in particular, tend to be very large and complex, and with the development of a new generation of detectors are growing explosively in number (Kuhlbrandt, 2014 ▸; Subramaniam *et al.*, 2016 ▸). A highlight for me as a card-carrying crystallographer, at the recent conference of the Asian Crystallographic Association (AsCA) in Hanoi, was to hear a beautiful account by Wah Chiu (Baylor College of Medicine, USA) of the ways in which cryo-EM map and model quality can now be assessed.

But science does not stand still, and these three principal structure determination methods are increasingly being complemented by data from other sources (Sali *et al.*, 2015 ▸), such as small-angle X-ray scattering (SAXS) and other solution scattering approaches. These help to expand the reach of structural biology into more complex systems, and it is important that these data, too, should be preserved. For these and other complementary methods there are difficult questions to be resolved. What are the key data that should be archived, and what metadata need to be captured with the experimental data if they are to be useful to other researchers?

A forthcoming article by Kroon-Batenburg *et al.* in this journal (Kroon-Batenburg *et al.*, 2017 ▸) highlights some of these issues. With the vastly expanded capacity of modern electronic media it is timely to ask whether raw crystallographic data files (the real primary data) could or should be archived in repositories where they can be accessed by other researchers. The advantages are many. With improved processing methods, better structures, at higher resolution, may be obtained. Other crystal phenomena such as diffuse scattering (often ignored in the pursuit of atomic structural models) could give new information on dynamics. ‘Pathological’ data sets that the original researchers had given up on could be reprocessed and might possibly bring valuable new structural information; we might bring to life a few of the skeletons that adorn our closets! The article by Kroon-Batenburg *et al.* is the latest update from a Working Group set up by the IUCr in 2011 to consider the practicalities of raw diffraction data deposition, and follows earlier papers on the topic, published in *Acta Crystallographica Section D* in 2014 (Terwilliger, 2014 ▸). It considers the present options for archiving raw data, and focuses particularly on the need for appropriate metadata to accompany the primary data if these data are to be truly useful into the future.

I am sure I am not alone in having in my office old nine-track magnetic tapes, DAT tapes and other media containing raw data sets from the past, none of them readable now as technologies become outdated. Science will be the poorer if our primary experimental data are lost, as some of these now are, and in the spirit of open science I consider these to be challenges that really must be addressed.
